# Calcium signalling in *Drosophila* photoreceptors measured with GCaMP6f

**DOI:** 10.1016/j.ceca.2017.02.006

**Published:** 2017-07

**Authors:** Sabrina Asteriti, Che-Hsiung Liu, Roger C. Hardie

**Affiliations:** Cambridge University, Department of Physiology Development and Neuroscience, Downing Street, Cambridge, CB2 3EG, UK

**Keywords:** TRP, transient receptor potential, InsP_3_, inositol (1,4,5) trisphosphate, IP_3_R, InsP_3_ receptor, PLC, phospholipase C, DPP, deep pseudopupil, R, rhodopsin, M, metarhodopsin, Phototransduction, TRP channels, Na/Ca exchanger, InsP_3_, Phospholipase C, Calcium imaging, Genetically encoded calcium indicators

## Abstract

*Drosophila* phototransduction is mediated by phospholipase C leading to activation of cation channels (TRP and TRPL) in the 30000 microvilli forming the light-absorbing rhabdomere. The channels mediate massive Ca^2+^ influx in response to light, but whether Ca^2+^ is released from internal stores remains controversial. We generated flies expressing GCaMP6f in their photoreceptors and measured Ca^2+^ signals from dissociated cells, as well as *in vivo* by imaging rhabdomeres in intact flies. In response to brief flashes, GCaMP6f signals had latencies of 10–25 ms, reached 50% *F*_max_ with ∼1200 effectively absorbed photons and saturated (*ΔF/F_0_* ∼ 10–20) with 10000–30000 photons. In Ca^2+^ free bath, smaller (*ΔF/F_0_* ∼4), long latency (∼200 ms) light-induced Ca^2+^ rises were still detectable. These were unaffected in InsP_3_ receptor mutants, but virtually eliminated when Na^+^ was also omitted from the bath, or in *trpl;trp* mutants lacking light-sensitive channels. Ca^2+^ free rises were also eliminated in Na^+^/Ca^2+^ exchanger mutants, but greatly accelerated in flies over-expressing the exchanger. These results show that Ca^2+^ free rises are strictly dependent on Na^+^ influx and activity of the exchanger, suggesting they reflect re-equilibration of Na^+^/Ca^2+^ exchange across plasma or intracellular membranes following massive Na^+^ influx. Any tiny Ca^2+^ free rise remaining without exchanger activity was equivalent to <10 nM (*ΔF/F_0_* ∼0.1), and unlikely to play any role in phototransduction.

## Highlights

• Fruitflies generated expressing GCaMP6f in photoreceptors using the opsin promoter.
• Quantitative *in vivo* Ca^2+^ imaging in completely intact flies as well as dissociated cells.
• Ca^2+^ rise in Ca^2+^ free bath abolished in Na^+^/Ca^2+^ exchanger mutants.
• Ca^2+^ free rise due to re-equilibration of Na^+^/Ca^2+^ exchange following Na^+^ influx.
• No significant light-induced release of Ca^2+^ from internal stores.

## Graphical abstract

**Figure:**
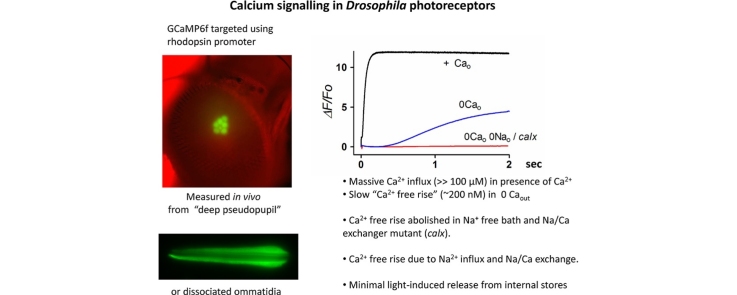

## 1 Introduction

Phototransduction in *Drosophila* is mediated by a G-protein coupled phospholipase C (PLC) signalling cascade [1], [2], [3]. All key elements of the transduction cascade from the visual pigment rhodopsin (Rh1) to the “light-sensitive” channels are localised within ∼30000 microvilli forming a light-guiding “rhabdomere”. Absorption of a single photon by one rhodopsin molecule results in a discrete electrical event (quantum bump), believed to reflect activation of PLC and ion channels within just one microvillus. The macroscopic current response to brighter light is the summation of multiple quantum bumps generated stochastically by absorption of photons across the microvillar population [4], [5]. There are two distinct light-sensitive channels in *Drosophila*: TRP (transient receptor potential), which is the prototypical and defining member of the TRP ion channel superfamily [6], [7], and a homologue, TRP-like, or TRPL [8], [9], both belonging to the TRPC subfamily. Although, both are cation channels permeable to Ca^2+^, TRP, which dominates the light-induced current (LIC) is particularly selective for Ca^2+^ (P_Ca_:P_Na_ ∼50:1), whilst TRPL has a more modest P_Ca_:P_Na_ of ∼4:1 [10], [11]. As well as mediating a major fraction of the LIC [12], Ca^2+^ influx via these channels plays critical positive and negative feedback roles at multiple downstream targets and is essential for rapid kinetics and light adaptation [13], [14].

Measurements using fluorescent Ca^2+^ indicators in dissociated *Drosophila* photoreceptors reveal that the Ca^2+^ signal in response to blue excitation light is dominated by massive Ca^2+^ influx via the light-sensitive channels [15], [16], [17]. Studies in larger flies using low affinity indicators show that Ca^2+^ levels in the microvilli reach near mM levels *in vivo* [18], and modelling suggests similar levels are reached in *Drosophila* [12], [19]. In Ca^2+^ free solutions there is a much smaller (submicromolar) and slower rise in fluorescence, the origin and role of which is controversial [17], [20], [21], [22]. Because InsP_3_ is presumably generated in large amounts in response to the blue excitation, InsP_3_-induced Ca^2+^ release from internal stores would seem the obvious explanation. However, using the high affinity ratiometric indicator INDO-1, this Ca^2+^ free signal was reported to be unaffected in null mutants of the only InsP_3_ receptor (IP_3_R) gene in the *Drosophila* genome [21]. Challenging this, Kohn et al. [22] reported that the Ca^2+^ free rise measured using the genetically encoded indicator GCaMP6f was substantially reduced following RNAi knockdown of the IP_3_R and proposed that InsP_3_-induced Ca^2+^ release played a critical role in phototransduction.

In the present study, we generated flies expressing GCaMP6f [23] in R1-6 photoreceptors under direct control of the Rh1 (*ninaE*) promoter. We performed measurements in dissociated ommatidia allowing control of extracellular solutions, and also *in vivo* from completely intact flies by imaging the rhabdomeres in the “deep pseudopupil” (DPP) [24], [25], [26]. By using 2-pulse protocols we provide data on the time course and intensity dependence of Ca^2+^ signals *in vivo* in response to physiologically relevant stimuli. We paid particular attention to the origin of the Ca^2+^ rise under Ca^2+^ free conditions, and found that it was unaffected in IP_3_R mutants, but strictly dependent upon both Na^+^ influx and Na^+^/Ca^2+^ exchanger activity. We conclude that any light-induced release from internal stores is minimal (<10 nM), slow, and unlikely to play any direct role in phototransduction.

## 2 Materials and methods

### 2.1 Flies

Flies (*Drosophila melanogaster*) were reared on standard medium [recipe in [27]] at 25 °C in a dark incubator. For dissociated ommatidia, newly eclosed (<2 h) adults were used; for *in vivo* deep pseudopupil measurements flies were 1–7 days old. GCaMP6f (cDNA obtained from Addgene) was cloned into the pCaSpeR4 vector which contains a mini-*w^+^* gene as transfection marker and the *ninaE* (*Rh1*) promoter that drives expression exclusively in photoreceptors R1-6. The final construct (*ninaE*-*GCaMP6f*) was injected into *w^1118^* embryos and transformants recovered on 2nd and 3rd chromosomes. The *ninaE*-*GCaMP6f* transgene was crossed into various genetic backgrounds including:

*trp^343^* – null mutant lacking TRP channels [28],

*trpl^302^* – null mutant lacking TRPL channels [9]

and *trpl^302^*;*trp^343^ –* double null mutant lacking all light-sensitive channels.

*norpA^P24^* – null mutant of PLC [29].

*calx^1^* – severe hypomorphic mutant of the Na^+^/Ca^2+^ exchanger (*calx*) with no detectable exchanger activity in the photoreceptors [30].

*ninaE-calx*/*CyO* – flies over-expressing a wild-type *calx* transgene under control of the Rh1-promoter [30].

(*l(3)itpr^90B.0^* – larval lethal null mutant of InsP_3_ receptor: referred to as *itpr* [31].

To generate *ninaE-GCaMP6f* in whole eye IP_3_R null (*itpr*) mosaics:

*ninaE-GCaMP6f/Cy;FRT82B*, (*l(3)itpr^90B.0^/TM6:* were crossed to

*yw;P{w+, ey-Gal4,UAS-FLP}/CyO;P{ry+,FRT82B}P{w+ GMR-hid},3CLR/TM6* – Bloomington stock 5253. Non-*Cy* and non-*TM6* F1 then have *itpr* homozygote null mosaic eyes and *ninaE-GCaMP6f* [21], [32].

### 2.2 Electrophysiology

Whole-cell patch clamp recordings of photoreceptors from dissociated ommatidia from newly eclosed adult flies of either sex were performed as previously described [e.g. 33] on an inverted Nikon microscope (Nikon UK). Standard bath contained (in mM): 120 NaCl, 5 KCl, 10 *N*-Tris-(hydroxymethyl)-methyl-2-amino-ethanesulphonic acid (TES), 4 MgCl_2_, 1.5 CaCl_2_, 25 proline and 5 alanine, pH 7.15. For Ca^2+^ free bath CaCl_2_ was omitted and 1 mM Na_2_EGTA added. The intracellular pipette solution was (in mM): 140 K gluconate, 10 TES, 4 Mg-ATP, 2 MgCl_2_, 1 NAD and 0.4 Na-GTP, pH 7.15. Chemicals were obtained from Sigma-Aldrich (Gillingham, UK). Recordings were made at room temperature (21 ± 1° C) at −70 mV (including correction for −10 mV junction potential) using electrodes of resistance 10–15 MΩ. Data were collected and analysed using an Axopatch 200 amplifier and pCLAMP v.9 or 10 software (Molecular Devices, Union City CA). Quantum bumps were analysed using Minianalysis software (Synaptosoft.com). Photoreceptors were stimulated via a green (522 nm) ultrabright light-emitting-diode (LED) controlled by a custom made LED driver; intensities were calibrated in terms of effectively absorbed photons by counting quantum bumps at low intensities.

### 2.3 GCaMP6f measurements

Fluorescence measurements were made as previously described [25], [34] on an inverted Nikon microscope (non-confocal) from dissociated ommatidia or *in vivo* by imaging the DPP in intact flies immobilised with low melting point wax in truncated plastic pipette tips. Excitation light (470 nm) was delivered from a blue power LED (Cairn Research UK) and fluorescence observed using 515 nm dichroic and OG515 long-pass filters. Fluorescent images were sampled and analysed at up to 500 Hz using an Orca 4 camera and HCImagelive software (Hamamtsu); but for most experiments fluorescence of whole ommatidia (via 40 x oil objective), or DPP (20 x air objective) was directly measured via a photomultiplier tube (Cairn Research UK), sampled at up to 2 kHz and analysed with pCLAMP software. Background fluorescence was subtracted using estimates from identical measurements from flies lacking fluorescent constructs, but with similar eye colour (most autofluorescence derives from screening pigment granules). Following each measurement the ommatidium/fly was exposed to intense, photo-equilibrating red (4 s, 640 nm ultra-bright LED) illumination to reconvert metarhodopsin (M) to rhodopsin (R), and allowed to dark adapt for at least one minute before the next measurement.

For Ca^2+^ free measurements, dissociated ommatidia (plated in standard bath) were briefly perfused with a Ca^2+^ free solution (0 Ca^2+^, 1 mM Na_2_EGTA see 2.2) or a Na^+^ and Ca^2+^ free solution in which NaCl was substituted for equimolar LiCl, KCl, CsCl or NMDGCl (1 mM K_2_EGTA and 4 mM MgCl_2_ also present). Ommatidia were individually perfused by a nearby (∼20 μm) puffer pipette and measurements made within ∼20–50 s of perfusion onset. Following M to R photoreconversion the cells were returned to normal (1.5 mM Ca^2+^) bath and dark-adapted for at least three minutes before the next measurement.

For 2-pulse experiments, green light was supplied by a green (λ_max_ 522 nm) LED (for dissociated ommatidia) or for the DPP by a “warm-white” power LED (Cairn Research UK) filtered by a GG 475 filter (resulting λ_max_ 546 nm). The green illumination was calibrated in terms of effectively absorbed photons by counting quantum bumps in whole-cell recordings or, for *in vivo* measurements from the DPP, by measuring the rate at which it converted M to R spectrophotometrically in the same set up, as previously described [26].

### 2.4 GCaMP6f calibration

Maximum and minimum fluorescence of GCaMP6f *in situ* was calibrated by exposing dissociated ommatidia to ionomycin (10 μM) and then perfusing alternately for several minutes with 10 mM K_2_EGTA 100 mM KCl 10 MOPS pH 7.2 (nominally 0 Ca^2+^) and 10 mM CaEGTA 100 mM KCl 10 MOPS (nominally 40 μM Ca^2+^) (solutions from Biotium Ca^2+^ calibration buffer kit). After background subtraction, *ΔF/F_0_* with the saturating 40 μM Ca^2+^ solution (*F*_max_) was 23.5 ± 1.52 (mean ± S.E.M. n = 8), which is close to the published *in vitro* value of 25 [35]. For estimating absolute cytosolic Ca^2+^ levels [Ca_i_] from *ΔF/F_0_* values, we assumed our *F*_max_ value of 23.5, the published K_d_ value (290 nM), and Hill slope (*n* = 2.7) [35] using the equation:

(1) [Ca_i_] = {K_d_. (*ΔF/F_0_)^1/n^}/{F_max_ − (ΔF/F_0_)}^1/n^*

## 3 Results

### 3.1 GCaMP6f Ca^2+^ signals under physiological conditions

**Fig. 1:**
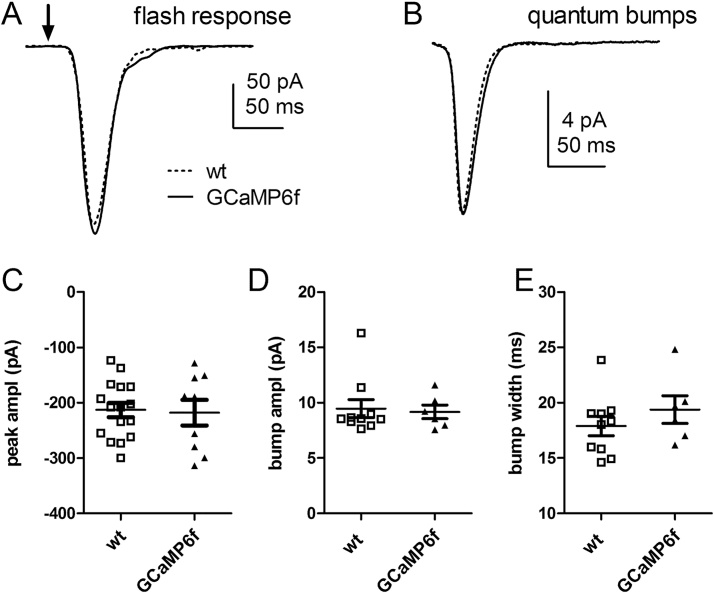
Light-induced currents from photoreceptors expressing GCaMP6f. (A) Whole-cell recordings of light-induced current responses to brief (1 ms) flashes (arrow), containing ∼100 effectively absorbed photons in a wild-type photoreceptor and a photoreceptor from *ninaE*-*GCamP6f* fly (each an average of 3 responses, voltage-clamped at −70 mV). (B) Averaged quantum bumps (after aligning rising phases) from wild-type (n = 10 cells, ∼40-60 bumps per cell) and GCaMP6f expressing photoreceptors (n = 6 cells). (C) Peak amplitudes to test flashes in wild-type (n = 16) and GCaMP6f expressing photoreceptors (n = 9) were indistinguishable (P = 0.846, 2-tailed *t*-test). (D & E) bump amplitudes and half-widths in wild-type (n = 10) and GCaMP6f expressing photoreceptors (n = 6) also showed no significant differences (P = 0.78 and 0.32 respectively).

**Fig. 2:**
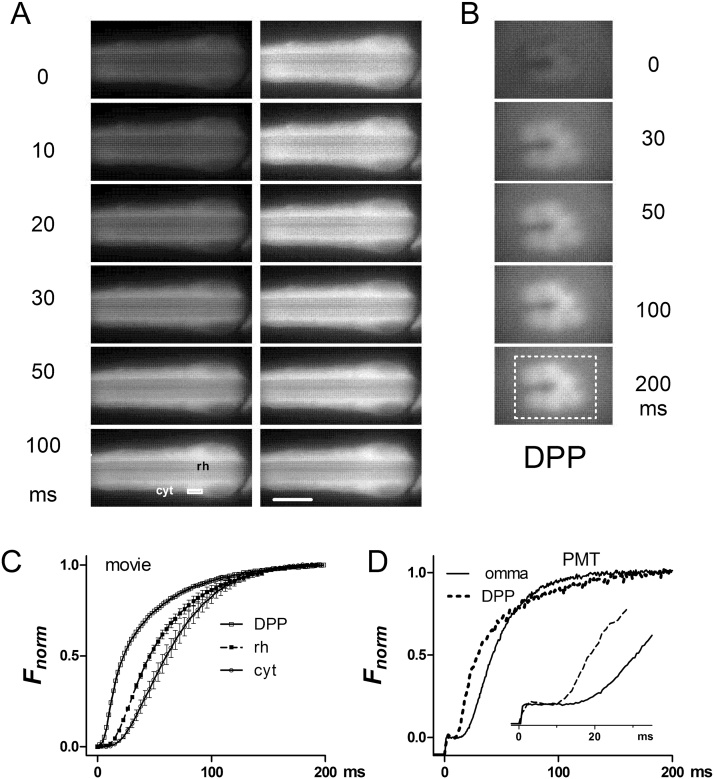
Live imaging of GCaMP6f. (A) GCaMP6f fluorescence in a dissociated ommatidium (distal end) from *ninaE*-*GCaMP6f* fly: 6 frames from 250 Hz movie (4 ms exposures; see Movie 1) at t = 0 to 100 ms after turning on blue excitation. Images on left are raw images with brightness and contrast adjusted with respect to the same (brightest) frame; images on right are the same but with brightness and contrast individually auto-adjusted. Scale bar 10 μm (×40 oil immersion objective). (B) Frames from a similar movie of GCaMP6f in rhabdomeres imaged in the deep pseudopupil (DPP) of an intact living *ninaE*-*GCaMP6f* fly (x 20 air objective). (C) Time-courses from movies (as in A & B). In dissociated ommatidia, regions of interest from rhabdomeres (rh) and cytosol (cyt) towards edge of the ommatidium (white box in A) were selected. For DPP a rectangle encompassing all 6 rhabdomeres was selected. Mean ± S.E.M. n = 5-6 ommatidia. Traces normalised to facilitate comparison of time course: maximum *ΔF/F_0_* values were in range 11–18 (see Fig. 3). (D) Normalised raw photomultiplier tube traces (PMT) sampled at 1 kHz, filtered at 0.5 kHz from *ninaE*-*GCaMP6f* flies. Representative single traces in response to supersaturating blue excitation are shown recorded from a dissociated ommatidium in normal bath (omma) and *in vivo* from the deep pseudopupil (DPP). Rising phases of the same traces shown in inset.

In order to monitor Ca^2+^ in *Drosophila* photoreceptors we expressed GCaMP6f directly under the control of the Rh1 opsin (*ninaE*) promoter (see methods), thereby driving expression specifically in the major photoreceptor class (R1-6). Whole-cell recordings from photoreceptors of these flies (*ninaE*-*GCaMP6f*) showed that their basic light responses were indistinguishable from wild-type (Fig. 1). Imaging of dissociated ommatidia revealed GCaMP6f diffusely distributed throughout the photoreceptors, with a weaker, though distinct signal in the rhabdomeres (Fig. 2, Fig. 5A). Other similarly sized GFP-tagged constructs (eg arrestin-GFP or PH-domain tagged GFPs) diffuse in and out of the microvilli within seconds [25], [26], so the weaker rhabdomere signal is presumably because of the small volume fraction of free cytosol in this membrane rich compartment rather than exclusion from rhabdomeres. High frame rate (100–500 Hz) movies showed a rapid increase in fluorescence (latency ∼10 ms) in response to the blue excitation, originating in the rhabdomeres and immediately adjoining cytosol, spreading outwards to the rest of the ommatidium with a lag of 10–20 ms to the outer edge of the cells (Fig. 2A,C; Movie 1). Fluorescence from the rhabdomeres can also be imaged in completely intact animals by focussing a low power objective in the depth of the eye to visualise the “deep pseudopupil” (DPP: Fig. 2 B) [24]. Rather than recording and analysing high frame rate movies, it is much more convenient to measure the fluorescence from either dissociated ommatidia or the DPP via a photomultiplier tube (PMT) using a portion of the field cropped by a diaphragm. Fig. 2D shows representative raw PMT traces of GCaMP6f fluorescence recorded in response to blue excitation light from a dissociated ommatidium and from the DPP in a completely intact fly. During a ∼10–20 ms latent period there is a clearly resolvable “pedestal”, which reflects GCaMP6f fluorescence corresponding to the initial dark-adapted Ca^2+^ concentration. After background correction (estimated from identical measurements in flies lacking GCaMP6f), the fluorescence rapidly rose to *ΔF/F_0_* values >10 within ∼200 ms. Although the signals were broadly similar, responses recorded from the DPP were faster than those recorded in dissociated ommatidia, probably because the DPP samples fluorescence predominantly from the rhabdomeres, whilst the ommatidium signal is dominated by cytosolic GCaMP6f. Apart from the pedestal (*F_0_*) and *F_max_* values, these signals *per se* are relatively uninformative: firstly because the blue excitation light is a super-saturating, non physiological stimulus, and secondly because GCaMP6f, with a K_d_ of 290 nM *in vitro* [35] should be saturated by Ca^2+^ concentrations in excess of ∼1–2 μM, whilst [Ca^2+^] in the photoreceptor is believed to reach values close to 1 mM in the rhabdomere and 10–50 μM in the cell body [17], [18].

**Fig. 3:**
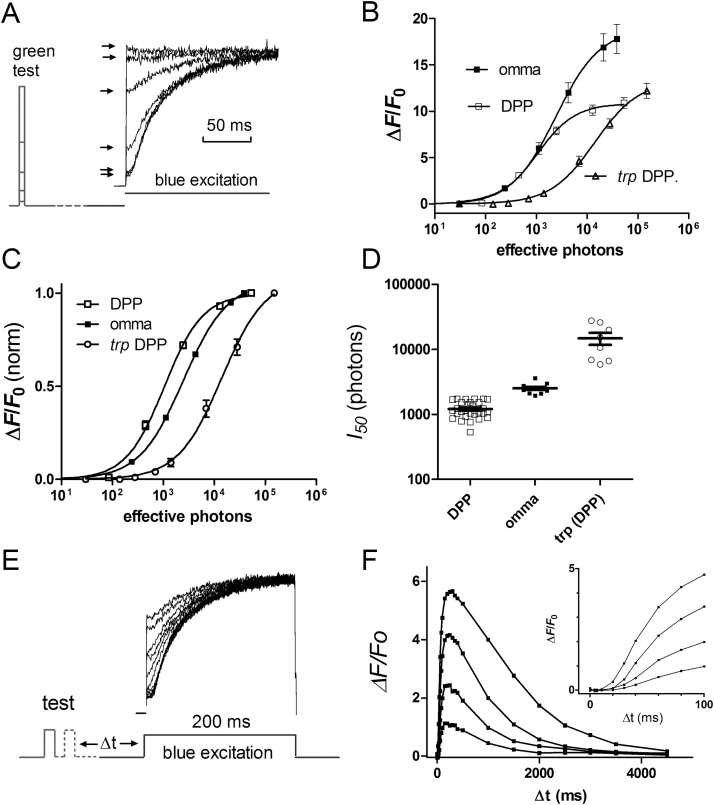
Intensity and time dependence of GCaMP6f signals using 2-pulse protocols. (A) 2 ms green flashes of different intensities were delivered 300 ms prior to blue excitation to measure GCaMP6f fluorescence from the DPP in completely intact *ninaE*-*GCaMP6f* flies. The pedestals (arrows) reflect the Ca^2+^ level in response to the green test flash (first response dark-adapted, i.e. without pre-flash). (B) resulting intensity dependence (*F*/log *I* function) after background correction, with respect to *F_0_* during dark adapted “pedestal”. Data from *trp* (DPP) also included, along with results from dissociated wild-type ommatidia. (C) Same data normalised. (D) Summary of sensitivity data: expressed in terms of number of effectively absorbed photons required to generate 50% *F*_max_. (E) 2-pulse protocol using brief (2 ms) flashes of the same intensity delivered with variable delay in order to measure the time course of GCaMP6f responses (*in vivo* from DPP). (F) Family of resulting impulse responses to flashes of increasing intensity (∼150,450,1250,5000 effective photons): inset shows the first 100 ms on a faster time base. In all experiments, after each blue excitation flash, M was reconverted to R by an intense, photo-equilibrating 4 s orange stimulus and the fly left in the dark for 1 min before the next test flash. Longer dark adaptation times result in slightly larger responses, but 1 min was chosen as a compromise to allow sufficient data collection (e.g. each time course trace in panel F required 24 repeated cycles- or ∼25 min – to record).

In order to measure the intensity dependence of the Ca^2+^ rise with respect to physiologically relevant intensities we used 2-pulse protocols in which brief (1–2 ms) calibrated flashes of green light were delivered 300 ms before measuring fluorescence (Fig. 3). The pedestals in these traces are still clearly resolvable but now reflect the Ca^2+^ level reached in response to the preceding test flash. The resulting *F*/log *I* functions had a steep intensity dependence with a threshold around 100 effectively absorbed photons, with 50% *F_max_* reached with intensities of ∼1000 photons (mean 1214 ± 64 S.E.M. n = 28) and saturating at intensities of 10000–30000 photons when measured *in vivo* from the DPP (Fig. 3 D). *F*/log *I* functions recorded from dissociated ommatidia were similar to those recorded *in vivo* from the DPP; however, the curves were slightly less steep and sensitivity ∼2-fold less (2516 ± 162 effective photons, n = 9 required to elicit 50% *F_max_*). The small difference in sensitivity may again reflect the predominantly rhabdomeric (DPP) vs cytosolic (dissociated ommatidium) source of the signals; but may also reflect the influence of the dissociation procedure, axial (DPP) as opposed to side-on (ommatidia) illumination, and/or the very different methods used to calibrate intensity; namely counting quantum bumps in whole-cell recordings from dissociated ommatidia, as opposed to measuring the metarhodopsin to rhodopsin photoisomerisation rate spectrophotometrically for the DPP (see methods 2.3).

We also expressed GCaMP6f in *trp^343^* null mutant flies lacking the dominant and more Ca^2+^ permeable of the two light-sensitive channels. Although maximum *ΔF/F_0_* values to saturating illumination were similar to wild-type (12.1 ± 2.3 n = 8; DPP measurements), *F*/log *I* functions determined with 2-pulse protocols were ∼10–20 x less sensitive, with 50% *F_max_* obtained with brief flashes containing 14800 ± 3100 (n = 8) effective photons (Fig. 3).

In order to measure the time course of Ca^2+^ responses we used similar 2-pulse protocols, this time presenting repeated brief (2 ms) flashes whilst varying the delay of the blue excitation light. Depending on intensity, Ca^2+^ rises were observed with latencies of ∼10–25 ms, peaking in 200–300 ms and then returning to baseline over a period of 2–4 s with a half time (t½) of ∼900 ms with dimmer test flashes (Figs. 3E,F and 6C,D). With brighter flashes, recoveries became somewhat slower, and with saturating flashes (∼30,000 photons) *ΔF/F_0_* remained high for 1–2 s, before recovering with a t½ of 3–4 s, still reaching baseline levels within ∼10 s (Fig. 3 and see also Fig. 7).

### 3.2 Ca^2+^ signals in Ca^2+^ free solutions

**Fig. 4:**
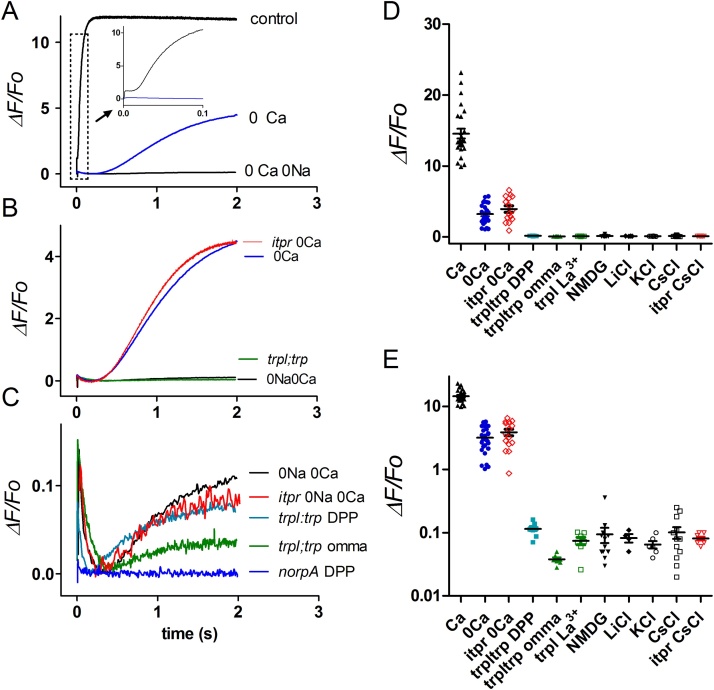
Dependence of GCaMP6f signals on Ca^2+^ and Na^+^ influx. (A–C): Fluorescence signals (PMT) measured from dissociated ommatidia expressing GCaMP6f. Traces are averages of 4–10 traces plotted as *ΔF/F_o_* (using *F_0_* values measured in Ca^2+^ free bath from same ommatidia). In control bath (1.5 mM CaCl_2_) values in excess of 10 were reached within 0.1 s (see inset of boxed area on expanded scale). In Ca^2+^ free (0 Ca^2+^, 1 mM Na_2_EGTA) bath there was a slow rise to ∼4, but with no detectable increase for at least 200 ms. In Ca^2+^ and Na^+^ free solutions (average of data recorded in 130 mM CsCl, LiCl, KCl or NMDG Cl, all with 1 mM K_2_EGTA 0 Ca^2+^ and 4 mM MgCl_2_), this slow response was almost eliminated leaving a slow rise to a *ΔF/F_o_* of only ∼0.1. (B) and (C) show same traces on different scales, with data from *trpl*;*trp* flies recorded in control bath solution (omma) and *in vivo* using the DPP, as well. Neither 0Ca or 0Ca 0Na responses were affected in null IP_3_R mutants (*itpr*). Note the initial transient decrease (origin uncertain), which is as large as the subsequent slow increase. These residual signals were both effectively eliminated in null PLC mutants (*norpA* DPP, n = 6). (D) and (E) Maximum *ΔF/F_o_* values (2 s after light onset) in dissociated ommatidia in different bath solutions, and *trpl*;*trp* measured *in vivo* using the DPP). Data from wild-type background unless otherwise indicated (*trpl*;*trp trpl* plus La^3+^, and *itpr*). Same data plotted on linear (D) and log_10_ scales (E).

**Fig. 5:**
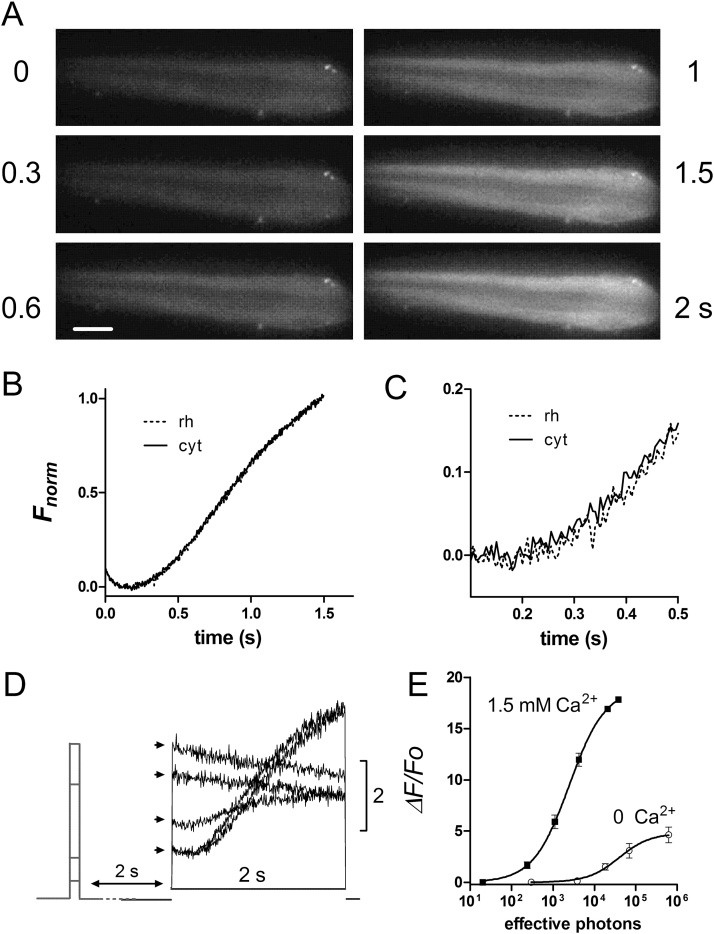
Image analysis and intensity dependence of Ca^2+^ free rises in dissociated ommatidia. (A) Six frames (0–2s) from a 100 Hz (10 ms exposure) movie (see Movie 2) of wild-type *ninaE*-*GCaMP6f* ommatidium in Ca^2+^ free bath (0 Ca^2+^, 1 mM EGTA 120 mM Na^+^). Brightness and contrast in all frames auto-adjusted to the final (brightest) frame. Bright spots towards distal (right) end of the ommatidium are autofluorescent pigment granules. Scale bar 10 μm. (B) Average time-courses (n = 6 ommatidia) from regions of interest covering rhabdomeres (rh) and cytosol (cyt) show near perfect overlap. Traces normalised to facilitate comparison of time-course: maximum *ΔF/F_0_* values were in range 2–5. (C) Rising phase on faster time base. (D) PMT fluorescence traces from a wild-type *ninaE*-*GCaMP6f* ommatidium, perfused with Ca^2+^ free bath (1 mM EGTA) for ∼30s. Brief (10 ms) green flashes of increasing intensity (0, 4000, 20000, 70000 and 600000 effective photons) were presented 2 s before the 2 s blue excitation. The instantaneous fluorescence “pedestals” (arrows) reflects the Ca^2+^ level reached in response to the green flashes. (D) Resulting *F*/log *I* function (mean ± S.E.M. n = 4) compared to data from ommatidia in normal bath (1.5 mM Ca^2+^ replotted from Fig. 3).

In dissociated ommatidia perfused with Ca^2+^ free solutions (0 Ca^2+^, 1 mM EGTA) there is a smaller and slower rise in Ca^2+^, the source and role of which is controversial [16], [17], [20], [21], [22]. In *ninaE*-*GCaMP6f* flies, this “Ca^2+^ free” signal can be recorded with excellent signal-to-noise ratio and reached *ΔF/F_o_* values of ∼1-6 (mean ∼3.2 ± 0.3 n = 24) after 2 s, which is now well within the dynamic range of GCaMP6f. Importantly, there was no detectable rise until at least ∼200 ms after light onset (Fig. 4), which would be too slow to have any influence on the rising phase of the electrical light response. In high frame rate movies (100–200 Hz), the Ca^2+^ appeared to rise more or less simultaneously across the ommatidium without any indication of a localised initial “release”, although the slow time course means that short time differences (10–20 ms) would not be reliably resolved (Fig. 5A–C; Movie 2). Assuming a resting baseline [Ca^2+^]_i_ of 50 nM in Ca^2+^free bath [17], a K_d_ for GCaMP6f of 290 nM, and Hill slope of 2.7 [35], the maximum *ΔF/F_o_* values would be equivalent to a modest rise to ∼100–210 nM Ca^2+^. We measured the intensity dependence of the Ca^2+^ free response using 2-pulse paradigms, presenting brief (10 ms) calibrated flashes 2 s before the blue excitation. Now, approximately 40000 effectively absorbed photons (>1 per microvillus) were required to elicit a 50% rise, which is ∼20–40 x more than for responses in physiological (Ca^2+^ containing) solutions or *in vivo* (Fig. 5D,E).

GCaMP6f signals in Ca^2+^ free bath were also characterised by an initial small transient decrease in fluorescence (*ΔF*/*F_0_* ∼ 0.1). The source of this is uncertain [22], but it was absent in *norpA^P24^* null PLC mutants (Fig. 4C), and had a similar time course to the pH drop measured using pH-sensitive dyes [34]. Because GCaMP6f fluorescence, like other GFP-based probes, is suppressed at acid pH (Hardie R.C. unpublished), one possibility is that it reflects pH sensitivity of GCaMP6f fluorescence in response to protons released by the PLC reaction[34].

Light-induced release from internal (InsP_3_-sensitive) Ca^2+^ stores would seem the most obvious explanation for the Ca^2+^ free signal; however, we found that it was completely unaffected in null mutants (mosaics) of the only InsP_3_ receptor gene (*itpr*) in the *Drosophila* genomes (Fig. 4B). This is in agreement with a previous study using Ca^2+^ indicator dyes in *itpr* mutants [21], but contrary to a recent study using GCaMP6f, where the Ca^2+^ rise in Ca^2+^ free solution was reported to be attenuated following IP_3_R RNAi knockdown [22]. It is difficult to reconcile these apparently contradictory results; however, we note that the signals we recorded in Ca^2+^ free solutions were slower than those reported by these authors in control ommatidia, but had similar time-courses to their responses in *IP_3_R*-*RNAi* flies. These authors used whole bath perfusion with a lower concentration of EGTA (0.5 mM) than our standard Ca^2+^ free solution (1 mM EGTA). We therefore also repeated measurements using 0.5 mM EGTA both in puffer pipettes and also after whole bath perfusion, but again in every case (n = 17 ommatidia in 6 flies) only slow (∼200 ms latency) responses were observed. Very occasionally (<5% of more than 100 ommatidia) we did see a more rapid Ca^2+^ signal; however, it was immediately clear that this was due to failure to adequately perfuse the ommatidium with Ca^2+^ free solution (e.g. a blocked puffer pipette). We can only speculate that a similar explanation may account for the rapid signals reported by Kohn et al. [22].

### 3.3 GCaMP6f signals in Ca^2+^ and Na^+^ free solutions

In an early study using the ratiometric indicator INDO-1 we reported that extracellular Na^+^ was required in order for a significant light-induced rise of cytosolic Ca^2+^ in Ca^2+^ free solutions [17]. Here we confirmed and extended this finding using GCaMP6f. Following perfusion of dissociated ommatidia by puffer pipette with EGTA buffered Ca^2+^ free and Na^+^ free solution, the initial transient decrease in fluorescence remained, but the subsequent rise was almost eliminated (Fig. 4). This was true whether Na^+^ was substituted for a range of similarly permeant monovalent cations (Li^+^ Cs^+^ or K^+^) or an essentially non-permeant cation (NMDG^+^).

Previously we suggested that the requirement of extracellular Na^+^ for a substantial Ca^2+^ free rise might reflect re-equilibration of Na^+^/Ca^2+^ exchange following the massive Na^+^ influx associated with these stimuli [17]. However, Cook and Minke [20] argued that only a Na^+^ gradient, and not Na^+^ influx, was necessary and suggested some other Na^+^ dependent process was required for release from internal stores. To test the requirement for Na^+^ influx, as opposed to a Na^+^ gradient we expressed GCaMP6f in *trpl*;*trp* double null mutants lacking all light-sensitive channels [9], [10], and hence all light-induced Na^+^ and Ca^2+^ influx, irrespective of the extracellular solution. Now, even in the presence of normal external Na^+^ and Ca^2+^, the GCaMP6f signal was at least as severely reduced as in wild-type ommatidia bathed in Ca^2+^ and Na^+^ free solutions, leaving again a tiny slow rise to a maximum *ΔF/F_o_* of <0.1 after 1–2 s (Fig. 4). In *trpl*;*trp* mutants, measurements without Ca^2+^ or Na^+^ influx could also be made *in vivo* from completely intact flies by measuring GCaMP6f fluorescence from the DPP. Even after prolonged (>3 h) dark adaptation, these measurements yielded similar results, with at most a tiny rise similar to that recorded in dissociated ommatidia (Fig. 4C).

We considered the possibility that the lack of a significant Ca^2+^ rise in *trpl*;*trp* mutants might be because potential light-sensitive internal Ca^2+^ stores were permanently depleted due the chronic lack of a Ca^2+^ influx pathway in the double mutant. To test for this we recorded GCaMP6f signals from ommatidia in *trpl* mutants in physiological solutions. The light responses in *trpl* are mediated exclusively by TRP channels and although almost indistinguishable from wild-type under physiological conditions, can be completely blocked by the TRP channel blocker, La^3+^ [9]. Correspondingly, the Ca^2+^ influx GCaMP6f signal prior to La^3+^ application in *trpl* flies was similar to wild-type (*ΔF/F_o_ > *10); however, following perfusion with La^3+^ (100 μM, 20–30 s application by puffer pipette) in the same ommatidia, only a tiny slow rise similar to that seen in Ca^2+^ and Na^+^ free solutions, or in *trpl*;*trp* double mutants remained (Fig. 4D,E).

Although it seemed reasonable to suspect that the tiny residual rise in *trpl*;*trp* or Na^+^ and Ca^2+^ free solutions might finally represent the rise due to InsP_3_-induced Ca^2+^ release, even this signal was still retained in ommatidia from *itpr* null mosaic eyes (Fig. 4C–E). The origin of this residual signal therefore remains uncertain. Given that it is of similar size to the initial transient decrease, it might also represent relaxation of this transient, and one cannot be confident that it still reflects a Ca^2+^ signal. Both the transient decrease and the subsequent slow rise/relaxation do however, seem to be PLC-dependent as neither signal could be detected in the null PLC mutant *norpA^P24^* (Fig. 4C).

In summary, these results indicate that Na^+^ influx, and not simply extracellular Na^+^, is required for a significant light-induced rise in Ca^2+^ in Ca^2+^ free solutions. The maximum residual fluorescence increase in the absence of Na^+^ or Ca^2+^ influx (*ΔF/F_o_* ∼ 0.1) would reflect a Ca^2+^ rise of the order of ∼10 nM. This signal developed slowly, was only detectable with very bright stimuli, was unaffected by the IP_3_R null mutation, and because of its tiny size one cannot even be confident it represents a Ca^2+^ signal.

### 3.4 Genetic manipulation of the Na^+^/Ca^2+^ exchanger

**Fig. 6:**
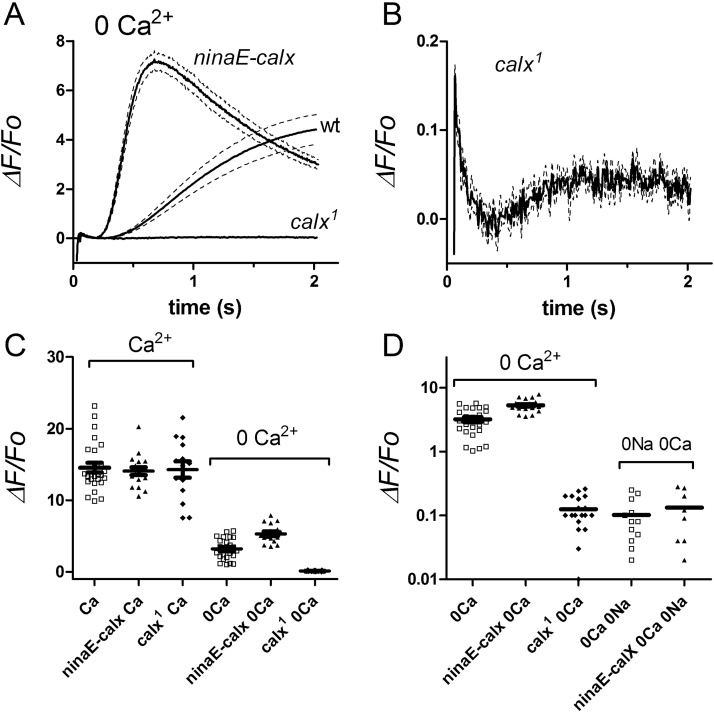
Effect of Na^+^/Ca^2+^ exchange on GCaMP6f signals in Ca^2+^ free solutions. (A) GCaMP6f fluorescence traces in response to blue excitation in 0 Ca^2+^ bath (1 mM EGTA) in dissociated ommatidia expressing GCaMP6f in wild-type, *calx^1^* mutants lacking exchanger activity and in *ninaE-calx* flies over-expressing the exchanger. Traces are mean ± S.E.M. n = 6-8 ommatidia. (B) *calx^1^* GCaMP6f trace (mean ± S.E.M. n = 6) replotted at high gain. (C) maximum *ΔF/F_o_* values (after 2 s) from wild-type, *calx^1^* and *ninaE-calx* backgrounds in both normal bath (Ca^2+^) and Ca^2+^ free bath (0 Ca^2+^) with normal Na^+^. (D) wild-type, *calx^1^* and *ninaE-calx* Ca^2+^ free *ΔF/F_0_* values replotted on log_10_ plot along with 0 Ca^2+^ 0 Na^+^ data (Cs^+^ substitution) from wild-type and *ninaE-calx.*

**Fig. 7:**
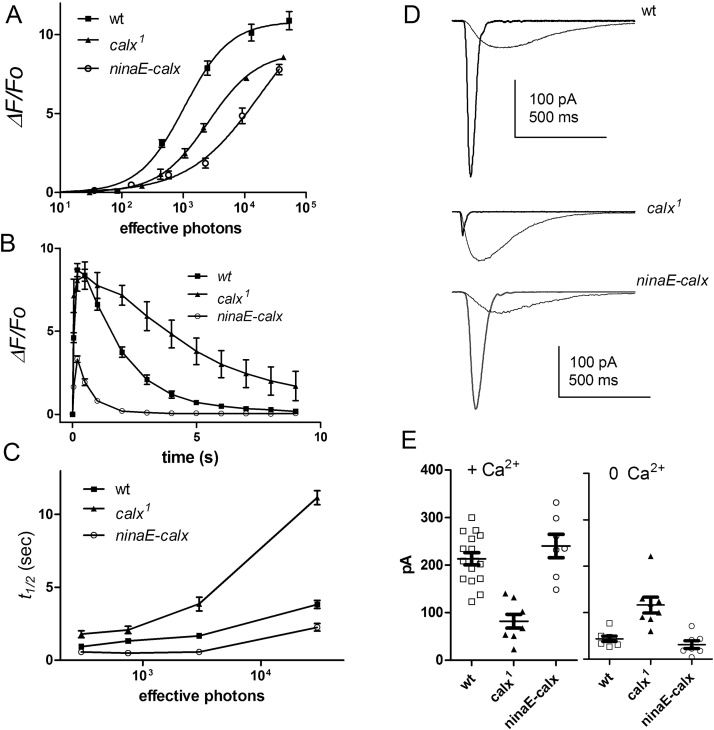
Effects of Na^+^/Ca^2+^ exchanger expression under physiological conditions. (A) GCaMP6f *F*/log *I* functions determined *in vivo* using 2-pulse DPP protocols (as in Fig. 3) in wild-type background (replotted from Fig. 3), *calx^1^* mutants (n = 7) and *ninaE-calx* flies (n = 5) over-expressing the exchanger. (B) Time courses of responses to brief 2 ms flashes containing ∼3000 effective photons (DPP 2-pulse data: mean ± S.E.M. n = 5−6 flies) in wild-type, *calx^1^* and *ninaE-calx.* (C) Time to 50% recovery (t½) of GCaMP6f signal as a function of intensity of flash (mean ± S.E.M. n = 4-9 flies) in wild-type, *calx^1^* and *ninaE*-*calx* flies (from time courses as in B). (D) Whole-cell recordings of light-induced currents in response to 1 ms flashes containing ∼100 effective photons in normal bath (rapid responses) and in Ca^2+^ free (1 mM EGTA) bath in wild-type, *calx^1^* and *ninaE-calx* photoreceptors (averages of 3 responses). (E) Summary of data: peak amplitudes in normal bath (left, + Ca^2+^) and Ca^2+^ free bath (right, 0 Ca^2+^). Ca^2+^ free responses were significantly (P < 0.001) larger in *calx^1^* mutants, and slightly (though not significantly: P = 0.23) decreased in *ninaE-calx*; whilst *calx^1^* responses were significantly (P < 0.001) smaller than wild-type in the presence of Ca^2+^.

If the normal Ca^2+^ free rise is due to re-equilibration of Na^+^/Ca^2+^ exchange following Na^+^ influx [17], we predicted that the rise should be prevented or reduced in mutants of the exchanger (encoded by the *calx* gene). We therefore expressed GCaMP6f in the severe *calx* hypomorph, *calx^1^*, which has no detectable exchanger activity [30]. As predicted, on perfusion with EGTA buffered Ca^2+^ free solution we were no longer able to detect any light-induced increase in GCaMP6f fluorescence in *calx^1^* mutants beyond a tiny residual signal similar to that seen in Ca^2+^ and Na^+^ free solutions in wild-type backgrounds (Fig. 6). By contrast, the Ca^2+^ rise *in vivo* (DPP) or from dissociated ommatidia in normal bath (i.e. 1.5 mM Ca^2+^) in *calx^1^* flies was broadly similar to that in wild-type flies; however, reflecting the pivotal role of the exchanger in Ca^2+^ extrusion, recovery to baseline in the dark was much slower, particularly following brighter flashes (Fig. 7B–D). Sensitivity (from *in vivo* 2-pulse *F*/log *I* functions) was also approximately 2–3 x lower than in wild-type (Fig. 7A).

In order to exclude the possibility that the loss of signal in Ca^2+^ free solutions was due to profound loss of responsivity, we made whole-cell recordings from *calx^1^* mutants. As previously reported [30], in normal physiological Ringer (1.5 mM Ca^2+^), *calx^1^* mutants showed an attenuated LIC, presumably due to the inhibitory effects of Ca^2+^. However, in Ca^2+^ free solutions, responses in *calx^1^* were actually significantly larger than in wild-type (Fig. 7E,F). We also considered the possibility that a potential Ca^2+^ free rise in *calx* mutants was masked by a higher resting Ca^2+^ level. However, with respect to *F_0_* in Ca^2+^ free solutions, maximum *ΔF/F_0_* values in *calx* flies in the presence of Ca^2+^ were similar to wild-type values (Fig. 6). Assuming *F_max_* was saturated in both cases, this means that *F_0_*–and hence resting Ca^2+^ in Ca^2+^ free solutions – was also similar in wild-type and *calx*.

We also expressed GCaMP6f in *ninaE-calx* flies, which over-express a wild-type *calx* transgene in photoreceptors R1-6, resulting in a 5–8 fold increase in Na^+^/Ca^2+^ exchanger activity [30]. We reasoned that if the light-induced Ca^2+^ rise in Ca^2+^ free solutions was due to re-equilibration of the exchanger in response to Na^+^ influx, it would be paradoxically accelerated in these flies; but if the rise was due to release from intracellular stores, then increasing the exchanger activity would only suppress the observed rise, because any released Ca^2+^ would be more rapidly extruded from the cell. Strikingly, in these flies the rise in Ca^2+^ in EGTA buffered Ca^2+^ free bath was indeed dramatically accelerated (Fig. 6A). The increase in rate of rise (∼7 fold) closely mirrored the increase in the exchanger current activity in *ninaE-calx* flies (5-fold faster, 8-fold larger in response to rapid Na^+^ substitution [30]), suggesting the rise may be directly attributable to exchanger activity. On average, the maximum *ΔF/F_0_* values reached were also significantly higher (5.3 ± 0.3 n = 17, P < 0.001 2-tailed *t*-test) than in otherwise wild-type flies. The accelerated Ca^2+^ free rise in *ninaE*-*calx* flies was still dependent upon Na^+^ influx because it was no longer seen after perfusion with Ca^2+^ and Na^+^ free solutions (Na^+^ substituted for Cs^+^), leaving instead a tiny residual rise similar to that in wild-type Ca^2+^ and Na^+^ controls (Fig. 6D). Finally, we asked whether the accelerated Ca^2+^ free rise might be explained by an unanticipated increase in responsivity; however, in whole-cell recordings from *ninaE-calx* photoreceptors in Ca^2+^ free solutions, sensitivity to light was if anything reduced compared to wild-type (Fig. 7 E,F), though this did not reach statistical significance on this sample (n = 7).

In marked contrast, under physiological conditions (using 2-pulse *in vivo* DPP measurements), over-expression of the exchanger in *ninaE*-*calx* flies had the predictable and opposite effect of suppressing Ca^2+^ rises in the *F*/log *I* function and also accelerating recovery to baseline (Fig. 7A–C); again supporting the dominant role of the exchanger in Ca^2+^ extrusion under physiological conditions.

In summary, our results show that the Ca^2+^ rise observed in Ca^2+^ free solutions is not only strictly dependent upon Na^+^ influx, but also strictly dependent on the activity level of the Na^+^/Ca^2+^ exchanger. Although we cannot completely exclude the possibility that the role of the exchanger is indirect, the fact that the Ca^2+^ free rise is accelerated in flies over-expressing the exchanger, yet still directly (and acutely) dependent upon Na^+^ influx strongly suggests that the rise can be attributed to the activity of the exchanger in response to massive Na^+^ influx, as originally proposed [17].

## 4 Discussion

Although Ca^2+^ signals in *Drosophila* photoreceptors were first studied over 20 years ago using Ca^2+^ indicator dyes [15], [16], [17], only one, recent study had used genetically encoded Ca^2+^ indicators [22]. These authors measured signals from dissociated ommatidia using the Gal4-UAS system [36], combining UAS-GCaMP6f with GMR-Gal4, which drives expression throughout the retina including all photoreceptor classes as well as accessory cells such as pigment and cone cells [37]. GMR-Gal4 expression also causes significant abnormalities in photoreceptor structure and physiology (Bollepalli M, Kuipers M, Liu C-H, Asteriti S, Hardie RC unpublished results)[38]. In the present study, we generated flies in which GCaMP6f expression was driven directly via the Rh1 (*ninaE*) promoter ensuring exclusive expression in R1-6 photoreceptors with wild-type morphology and physiology. The excellent signal-to-noise ratio of recordings in *ninaE*-*GCaMP6f* flies was distinctly superior to that in GMR-Gal4/UAS-GCaMP6f flies, and in many cases the maximum *ΔF/F_0_* ratio approached or exceeded 20 (cf ∼3 using GMR-Gal4/UAS-GCaMP6f [22]). This is close to the maximum value (23.5) determined by *in situ* calibrations (see methods 2.4) or *in vitro* [35]. Although the blue excitation light used for measuring GCaMP6f fluorescence is a super-saturating stimulus, 2-pulse paradigms allowed sensitive and accurate measurements of intensity and time dependence of signals in response to stimuli in the physiological range. Recordings *in vivo* from the DPP of intact flies are simple to perform and can be readily maintained over many hours, making this approach a valuable, and completely non-invasive tool for assessing *in vivo* photoreceptor performance. Even in the more vulnerable dissociated ommatidia preparation, multiple repeatable measurements could be made for up to at least an hour from the same ommatidium as long as metarhodopsin was reconverted to rhodopsin by long wavelength light after each measurement.

### 4.1 Ca^2+^ signals under physiological conditions

*In vivo* (DPP) or in dissociated ommatidia bathed in physiological solutions, the GCaMP6f signal reached 50% *F*_max_ at intensities equivalent to ∼1000–2500 effectively absorbed photons. It is believed that the elementary single photon response (quantum bump) is generated by activation of Ca^2+^ permeable channels (TRP and TRPL) within a single microvillus and that the consequent Ca^2+^ rise in the affected microvillus reaches near mM levels [18], [19]. Because such levels inevitably saturate GCaMP6f (K_d_ 290 nM, saturating at 1–2 μM), to a first approximation the *ΔF/F_0_* values under physiological conditions are probably best interpreted as the proportion of microvilli “flooded” with Ca^2+^. In total, the rhabdomere contains ∼30000 microvilli, meaning that 50% *F*_max_ is reached when only ∼3–8% of the microvilli have been activated by a photon. This implies that the Ca^2+^ influx into a single microvillus must spread to at least the immediately neighbouring microvilli within the timeframe of the response. In *ninaE-calx* flies over-expressing the Na^+^/Ca^2+^ exchanger, or in *trp* mutants lacking the major Ca^2+^ permeable channel, 50% *F_max_* was only obtained with flashes containing ∼ 12000-15000 effective photons, which should activate ∼50% of the microvilli, suggesting that in these flies Ca^2+^ is largely prevented from spreading to neighbouring microvilli under the same conditions.

The dark-adapted “pedestal” level can be used to gain an estimate of the resting Ca^2+^ concentration in dissociated ommatidia (in physiological solutions) assuming *in vitro* calibration data [35]. With reference to *F_0_* measured in Ca^2+^ free solution in the same ommatidia, the mean dark-adapted value in normal bath was 0.77 ± 0.14 (mean ± S.E.M. n = 11), which would be equivalent to ∼80 nM (assuming K_d_ = 290 nM and *F_max_* 23.5 and Eq. (1) see methods 2.4). This value was significantly lower in *ninaE-calx* flies over-expressing the exchanger (0.19 ± 0.04 n = 11 equivalent to ∼50 nM) and higher in *calx^1^* mutants (1.94 ± 0.24 n = 14 equivalent to ∼120 nM).

The recovery of GCaMP6f fluorescence to baseline is likely to be a reasonably accurate reflection of the falling Ca^2+^ levels during response recovery, although the initial decrease (from initial ∼mM levels to low μM levels) will still be subject to saturation effects. With relatively dim flashes (up to ∼1000 effectively absorbed photons) the GCaMP6f signal in wild-type backgrounds fell to near baseline within ∼2–3 s with a half time (t ½) of ∼1 s (Figs. 3, 7). This is slower than the GCaMP6f off-rate (∼200 ms), and thus likely to approximate the true time-course of Ca^2+^ recovery. The recovery was significantly accelerated in *ninaE-calx* flies (∼500 ms), and slowed in *calx^1^* mutants (∼2 s increasing to >10 s following brighter flashes; Fig. 7), consistent with a dominant role of the Na^+^/Ca^2+^ exchanger in Ca^2+^ extrusion [30]. Nevertheless, even after bright flashes, given sufficient dark-adaptation time (∼30–60 s), resting [Ca^2+^] in *calx^1^* mutants fell to levels close to those in dark-adapted wild-type photoreceptors, reflecting either residual function of the exchanger in this hypomorphic mutant and/or alternative extrusion mechanism(s).

### 4.2 Origin of the Ca^2+^ rise under Ca^2+^ free conditions

The smaller signals recorded in Ca^2+^ free bath fall within the dynamic range of GCaMP6f and allow estimates of the absolute Ca^2+^ levels reached under these conditions (e.g. *ΔF/F_0_* of 6 corresponds to ∼200 nM). We used these signals to investigate the long disputed origin of the light-induced rise in cytosolic Ca^2+^ in Ca^2+^ free solutions. Originally, using INDO-1, we found that this Ca^2+^ free rise was dependent upon extracellular Na^+^ and suggested that the rise might be due to re-equilibration of Na^+^/Ca^2+^ exchange in response to the massive light-induced Na^+^ influx that persists under these conditions [17]. This was challenged by Cook and Minke [20] who confirmed the requirement of external Na^+^ for a significant Ca^2+^ rise in Ca^2+^ free solutions, Na^2+^, but reported that a rise still occurred in Ca^2+^ free bath in the presence of Na^+^ when the photoreceptors were voltage clamped at the Na^+^ equilibrium potential to prevent Na^+^ influx. They concluded that a Na^+^ gradient − but not influx − was required, that the Ca^2+^ free rise reflected release from internal stores, and that the requirement of extracellular Na^+^ reflected involvement of some other Na^+^ dependent process, such as Na/H transport. But how this might affect release of Ca^2+^ from intracellular stores is far from clear. A more recent study from the same lab reported that the Ca^2+^ free rise was attenuated following RNAi knockdown of the IP_3_R [22]. However, this is difficult to reconcile with an earlier study using INDO-1, where the rise was found to be unaffected in null IP_3_R mosaic eyes [21] and confirmed again here using GCaMP6f (Fig. 4).

In the present study we used a variety of approaches to investigate the source of this Ca^2+^ free signal further. We first confirmed that it was all but abolished in the absence of external Na^+^, whether substituted for Li^+^, Cs^+^, K^+^ or NMDG^+^. Importantly, we found that the rise was also effectively eliminated in *trpl*;*trp* double mutants both *in vivo* and in dissociated ommatidia despite the presence of normal extracellular solutions containing both Na^+^ and Ca^2+^. Although it might be argued that, for some reason, PLC activity (and hence InsP_3_ generation) was compromised in *trpl*;*trp* mutants, convincing evidence indicates that net PLC activity is in fact greatly enhanced in *trpl*;*trp* due to the lack of Ca^2+^ and PKC dependent inhibition of PLC. Thus the rate and intensity dependence of PIP_2_ hydrolysis, measured using GFP-tagged PIP_2_ binding probes are greatly enhanced in *trpl*;*trp* mutants [26], as are the PLC-induced photomechanical contractions [39], and the acidification due to the protons released by the PLC reaction [34]. Overall, therefore these results strongly suggest that Na^+^ influx is indeed required for the Ca^2+^ free rise. Crucially, the involvement of the Na^+^/Ca^2+^ exchanger in this rise was confirmed by finding that it was essentially eliminated in an exchanger mutant (*calx^1^*), but greatly accelerated in *ninaE-calx* photoreceptors over-expressing the exchanger (Fig. 6).

The question remains, how Na^+^/Ca^2+^ exchanger activity could generate such a sizeable Ca^2+^ signal (∼100–200 nM) in cells perfused with EGTA buffered solutions, when free Ca^2+^ in the bath should be reduced to low nM levels. We do not have an unequivocal answer to this, and assuming the standard equation for the Na^+^/Ca^2+^ exchange equilibrium

(2) $$\left[Ca_{i}\right]=\left[Ca_{o}\right]\frac{{\left[Na_{i}\right]}^{3}}{{\left[Na_{o}\right]}^{3}}e^{\frac{EF}{RT}}$$

it would seem difficult for reverse Na^+^/Ca^2+^ exchange to raise Ca^2+^ into the range we observed. However, at least three, not mutually exclusive factors might result in higher cytosolic Ca_i_ levels than predicted. Firstly, external Ca^2+^ might be relatively resistant to buffering in the intra-ommatidial space, and specifically the extremely narrow spaces between the microvilli or their bases, where the exchanger is believed to be localised [30], [40]. For example, with 500 nM Ca_o_ remaining, Eq. (2) predicts 130 nM Ca_i_ would be reached with 70 mM Na_i_, 110 mM Na_o_ and the cell depolarised to 0 mV (values that could realistically be reached with the huge inward Na^+^ currents flowing under these conditions). Although one might also expect Ca^2+^ influx via the light-sensitive channels at such Ca_o_ concentrations, experiments buffering external Ca^2+^ at different concentrations with EGTA showed that direct Ca^2+^ influx signals could only be detected once external Ca^2+^ was raised above ∼400 nM (Suppl. Fig. 1). Secondly, resting cytosolic Ca^2+^ concentration is determined not only by the exchanger, but also by any other Ca^2+^ fluxes, which might include tonic leakage from intracellular compartments such as endoplasmic reticulum (ER) or mitochondria. Massive Na^+^ influx would compromise the ability of the exchanger to counter any such fluxes. A third possibility is that, contrary to conventional dogma, the exchanger might also be expressed on intracellular membranes of endoplasmic reticulum or other Ca^2+^ containing compartments and that Na^+^ influx leads to re-equilibration of Na^+^/Ca^2+^ exchange across these.

Whatever the exact mechanism, our results indicate that the Ca^2+^ rise in Ca^2+^ free bath is strictly dependent upon both Na^+^ influx and the activity level of the Na^+^/Ca^2+^ exchanger, but unaffected in null IP_3_R mutants. Its time-course, with no detectable rise for ∼200 ms, also appears much too slow to play any role in initiating the light response, which has a latency of ∼10 ms and peaks within ∼100–200 ms in response to bright illumination even under Ca^2+^ free conditions [34][e.g. 34]. The residual GCaMP6f signal remaining in the absence of Na^+^ influx and/or in the absence of Na^+^/Ca^2+^ exchanger activity − whether achieved by Na^+^ substitution, *trpl*;*trp* or *calx* mutants − was also still observed in IP_3_R mutants and was so small that it is questionable whether it reflects a Ca^2+^ signal. Because of the rapid inhibition of PLC by Ca^2+^ influx under physiological conditions [13], [34], [41] any presumptive PLC-mediated Ca^2+^ release under physiological conditions would be even less. Together with a study in which we found no phototransduction defects in null IP_3_R mutants (Bollepalli M,Kuipers M, Liu C-H, Asteriti S, Hardie RC unpublished results), these results suggest that InsP_3_-induced Ca^2+^ release plays no significant role in *Drosophila* phototransduction.

## Conflict of interest

The authors declare no competing financial interests

## Author contributions

RCH conceived and designed the research, wrote the paper and performed and analysed Ca^2+^ imaging experiments. SA performed and analysed Ca^2+^ imaging and whole-cell electrophysiology experiments. C-HL performed molecular biology and fly genetics. SA and C-HL commented on the MS.

## Figures and Tables

**Figure:**
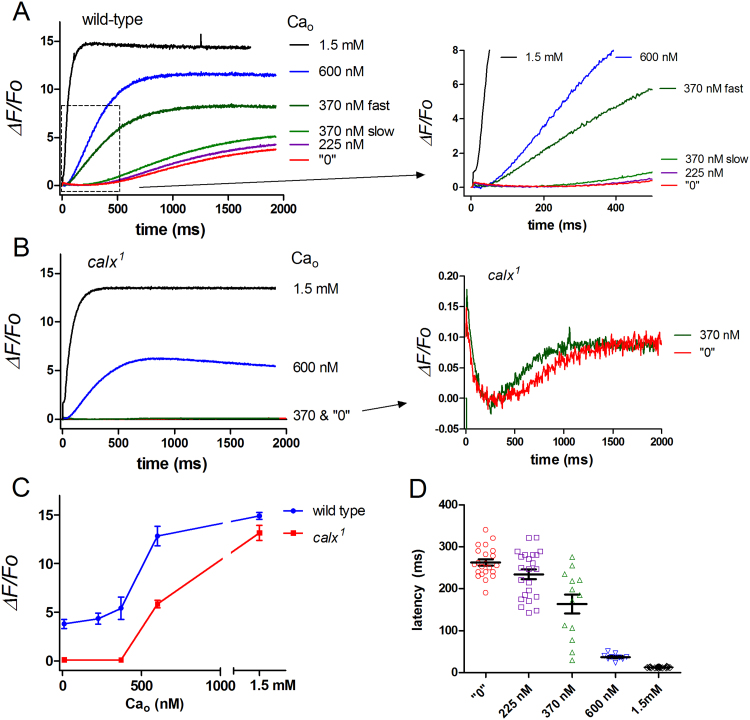


## References

[1] Montell C. (2012). *Drosophila* visual transduction. Trends Neurosci..

[2] Hardie R.C., Juusola M. (2015). Phototransduction in *Drosophila*. Curr. Opin. Neurobiol..

[3] Katz B., Minke B. (2009). *Drosophila* photoreceptors and signaling mechanisms. Front. Cell. Neurosci..

[4] Song Z., Postma M., Billings S.A., Coca D., Hardie R.C., Juusola M. (2012). Stochastic, adaptive sampling of information by microvilli in fly photoreceptors. Curr. Biol..

[5] Henderson S.R., Reuss H., Hardie R.C. (2000). Single photon responses in *Drosophila* photoreceptors and their regulation by Ca^2+^. J. Physiol. Lond..

[6] Hardie R.C., Minke B. (1992). The *trp* gene is essential for a light-activated Ca^2+^ channel in *Drosophila* photoreceptors. Neuron.

[7] Montell C., Rubin G.M. (1989). Molecular characterization of *Drosophila trp* locus, a putative integral membrane protein required for phototransduction. Neuron.

[8] Phillips A.M., Bull A., Kelly L.E. (1992). Identification of a *Drosophila* gene encoding a calmodulin-binding protein with homology to the trp phototransduction gene. Neuron.

[9] Niemeyer B.A., Suzuki E., Scott K., Jalink K., Zuker C.S. (1996). The *Drosophila* light-activated conductance is composed of the two channels TRP and TRPL. Cell.

[10] Reuss H., Mojet M.H., Chyb S., Hardie R.C. (1997). *In vivo* analysis of the *Drosophila* light-sensitive channels, TRP and TRPL. Neuron.

[11] Liu C.H., Wang T., Postma M., Obukhov A.G., Montell C., Hardie R.C. (2007). I*n vivo* identification and manipulation of the Ca^2+^ selectivity filter in the *Drosophila* Transient Receptor Potential Channel. J. Neurosci..

[12] Chu B., Postma M., Hardie R.C. (2013). Fractional Ca^2+^ currents through TRP and TRPL channels in *Drosophila* photoreceptors. Biophys. J ..

[13] Gu Y., Oberwinkler J., Postma M., Hardie R.C. (2005). Mechanisms of light adaptation in *Drosophila* photoreceptors. Curr. Biol..

[14] O'Tousa J.E. (2002). Ca^2+^ regulation of *Drosophila* phototransduction. Adv. Exp. Med. Biol..

[15] Peretz A., Suss-Toby E., Rom-Glas A., Arnon A., Payne R., Minke B. (1994). The light response of *Drosophila* photoreceptors is accompanied by an increase in cellular calcium: effects of specific mutations. Neuron.

[16] Ranganathan R., Bacskai B.J., Tsien R.Y., Zuker C.S. (1994). Cytosolic calcium transients: spatial localization and role in *Drosophila* photoreceptor cell function. Neuron.

[17] Hardie R.C. (1996). INDO-1 measurements of absolute resting and light-induced Ca^2+^ concentration in *Drosophila* photoreceptors. J. Neurosci..

[18] Oberwinkler J., Stavenga D.G. (2000). Calcium transients in the rhabdomeres of dark- and light-adapted fly photoreceptor cells. J. Neurosci..

[19] Postma M., Oberwinkler J., Stavenga D.G. (1999). Does Ca^2+^ reach millimolar concentrations after single photon absorption in *Drosophila* photoreceptor microvilli?. Biophys. J ..

[20] Cook B., Minke B. (1999). TRP and calcium stores in *Drosophila* phototransduction. Cell Calcium.

[21] Raghu P., Colley N.J., Webel R., James T., Hasan G., Danin M., Selinger Z., Hardie R.C. (2000). Normal phototransduction in *Drosophila* photoreceptors lacking an InsP_3_ receptor gene. Mol. Cell. Neurosci..

[22] Kohn E., Katz B., Yasin B., Peters M., Rhodes E., Zaguri R., Weiss S., Minke B. (2015). Functional cooperation between the IP3 receptor and phospholipase C secures the high sensitivity to light of *Drosophila* photoreceptors in vivo. J. Neurosci..

[23] Chen T.W., Wardill T.J., Sun Y., Pulver S.R., Renninger S.L., Baohan A., Schreiter E.R., Kerr R.A., Orger M.B., Jayaraman V., Looger L.L., Svoboda K., Kim D.S. (2013). Ultrasensitive fluorescent proteins for imaging neuronal activity. Nature.

[24] Franceschini N., Kirschfeld K. (1971). Les phenomenes de psedoupille dans l'oeil compose de *Drosophila*. Kybernetik.

[25] Satoh A.K., Xia H., Yan L., Liu C.H., Hardie R.C., Ready D.F. (2010). Arrestin translocation is stoichiometric to rhodopsin isomerization and accelerated by phototransduction in *Drosophila* photoreceptors. Neuron.

[26] Hardie R.C., Liu C.H., Randall A.S., Sengupta S. (2015). In vivo tracking of phosphoinositides in *Drosophila* photoreceptors. J. Cell Sci..

[27] Randall A.S., Liu C.H., Chu B., Zhang Q., Dongre S.A., Juusola M., Franze K., Wakelam M.J., Hardie R.C. (2015). Speed and sensitivity of phototransduction in *Drosophila* depend on degree of saturation of membrane phospholipids. J. Neurosci..

[28] Scott K., Sun Y.M., Beckingham K., Zuker C.S. (1997). Calmodulin regulation of *Drosophila* light-activated channels and receptor function mediates termination of the light response in vivo. Cell.

[29] Pearn M.T., Randall L.L., Shortridge R.D., Burg M.G., Pak W.L. (1996). Molecular, biochemical, and electrophysiological characterization of *Drosophila norpA* mutants. J. Biol. Chem..

[30] Wang T., Xu H., Oberwinkler J., Gu Y., Hardie R.C., Montell C. (2005). Light activation, adaptation, and cell survival Functions of the Na^+^/Ca^2+^ exchanger CalX. Neuron.

[31] Venkatesh K., Hasan G. (1997). Disruption of the IP3 receptor gene of *Drosophila* affects larval metamorphosis and ecdysone release. Curr. Biol..

[32] Stowers R.S., Schwarz T.L. (1999). A genetic method for generating *Drosophila* eyes composed exclusively of mitotic clones of a single genotype. Genetics.

[33] Hardie R.C., Martin F., Cochrane G.W., Juusola M., Georgiev P., Raghu P. (2002). Molecular basis of amplification in *Drosophila* phototransduction. Roles for G protein, phospholipase C, and diacylglycerol kinase. Neuron.

[34] Huang J., Liu C.H., Hughes S.A., Postma M., Schwiening C.J., Hardie R.C. (2010). Activation of TRP channels by protons and phosphoinositide depletion in *Drosophila* photoreceptors. Curr. Biol..

[35] Badura A., Sun X.R., Giovannucci A., Lynch L.A., Wang S.S. (2014). Fast calcium sensor proteins for monitoring neural activity. Neurophotonics.

[36] Brand A.H., Perrimon N. (1993). Targeted gene expression as a means of altering cell fates and generating dominant phenotypes. Development.

[37] Hay B.A., Maile R., Rubin G.M. (1997). P element insertion-dependent gene activation in the *Drosophila* eye. Proc. Natl. Acad. Sci. U. S. A..

[38] Kramer J.M., Staveley B.E. (2003). GAL4 causes developmental defects and apoptosis when expressed in the developing eye of *Drosophila melanogaster*. Genet. Mol. Res..

[39] Hardie R.C., Franze K. (2012). Photomechanical responses in *Drosophila* photoreceptors. Science.

[40] Oberwinkler J., Stavenga D.G. (2000). Calcium imaging demonstrates colocalization of calcium influx and extrusion in fly photoreceptors. Proc. Natl. Acad. Sci. U. S. A..

[41] Hardie R.C., Raghu P., Moore S., Juusola M., Baines R.A., Sweeney S.T. (2001). Calcium influx via TRP channels is required to maintain PIP_2_ levels in *Drosophila* photoreceptors. Neuron.

