# Supplementary figures and images for: Calcium signalling in Drosophila photoreceptors measured with GCaMP6f
Source: Cell Calcium. 2017 Jul;65:40–51. doi: 10.1016/j.ceca.2017.02.006 (PMC5472182; doi:10.1016/j.ceca.2017.02.006)

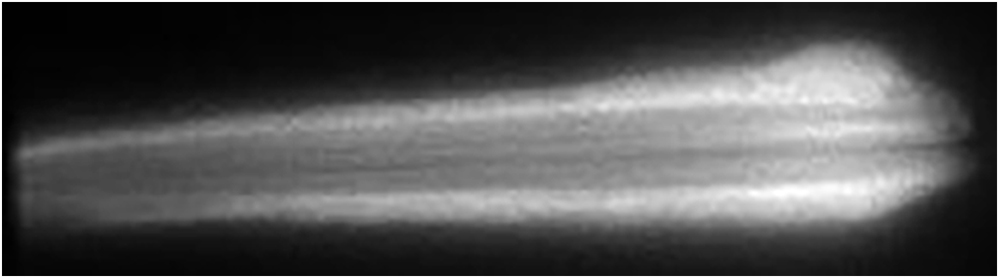

Supplement: Supplementary file 1 [file mmc1.jpg]

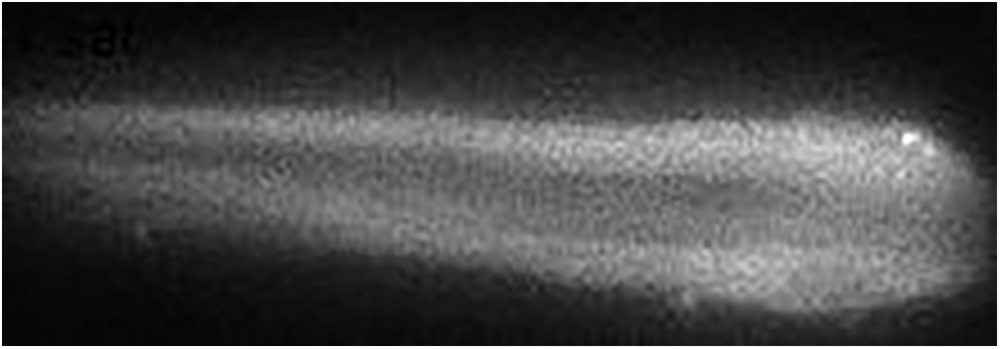

Supplement: Supplementary file 2 [file mmc2.jpg]
