# Supplementary material for: Calcium signalling in Drosophila photoreceptors measured with GCaMP6f
Source: Cell Calcium. 2017 Jul;65:40–51. doi: 10.1016/j.ceca.2017.02.006 (PMC5472182; doi:10.1016/j.ceca.2017.02.006)
Supplement: Supplementary file 3 [file mmc4.docx]

**Supplemental Figure 1 External Ca^2+^ dependence of light-induced GCaMP6f signals**

A &B) GCaMP6f fluorescence from dissociated ommatidia of wild-type (A) and *calx^1^* mutants (B). Ommatidia were perfused for 30s with solutions containing 1 mM free EGTA and different concentrations of Ca^2+^ to achieve a range of free Ca^2+^ concentrations (Ca_o_) from nominally Ca^2+^ free (“0” : i.e. 1mM EGTA, no added Ca) to 600 nM (5mM EGTA, 4 mM Ca). Control traces in normal bath (1.5 mM Ca^2+^) also shown. Each trace is the average from 4-8 ommatidia. Note that with 225 nM Ca_o_ and most ommatidia with 370 nM there was still a ~200-300 ms latency, but in a few ommatidia (3/13 ) at 370 nM and all ommatidia at 600 nM the latency shifted dramatically to less than 100 ms (mean ~40 ms at 600 nM; see inset on right on faster time base). In *calx^1^* mutants even at 370 nM, there was no Ca^2+^ rise beyond the residual ~0.1 signal of uncertain origin (n=7: right inset on expanded scale), but in every case a robust Ca^2+^ signal was seen with 600 nM Ca_o_. C) Maximum *ΔF/F_o_* values reached within 2 seconds as a function of external Ca^2+^ (mean ± S.E.M. n = 6 -16 ommatidia per data point). D) GCaMP6f fluorescence response latencies at different external Ca^2+^ concentrations (wild-type data only). Data were collected from a total of 54 wild-type ommatidia from 10 different flies (wt) and 19 *calx* mutant ommatidia (4 flies), and are independent from data in the main text. In all cases, ommatidia were bathed in normal bath (1.5 mM Ca^2+^) and perfused (by puffer pipette) for 30 s before excitation.

Together these data indicate that there is usually no detectable Ca^2+^ influx signal below 370 nM, whilst at 600 nM Ca_o_ , Ca^2+^ influx can invariably be clearly detected and distinguished from the Na//Ca exchanger dependent slow rise at lower Ca^2+^ concentrations. The rapid kinetics of responses with 600 nM Ca_o_ , which we attribute to Ca^2+^ influx, closely resemble the “Ca^2+^ free” traces of Kohn et al. 2015, consistent with our speculation that the bath in their experiments might still have been contaminated with Ca^2+^.

VIDEO legends

**Movie 1** 250Hz (4 ms per frame) wild-type ommatidium expressing *ninaE*-*GCaMP6f* in normal bath (1.5 mM Ca^2+^). Total duration of movie 200 ms.

**Movie 2** 100 Hz (10 ms per frame) wild-type ommatidium expressing *ninaE*-*GCaMP6f* in Ca free bath (1 mM EGTA). Total duration 2 sec.
